# 

**Published:** 1841-07

**Authors:** 


### WESTERN JOURNAL

OF

### MEDICINE AND SURGERY.

### COLLABORATORS.

---

Edward H. Barton, M. D. *Professor of the Theory and Practice of Medicine, in the Medical College of Louisiana.*

William J. Barbee, M. D. *Cincinnati, Ohio.*

George W. Bayless, M. D. *Demonstrator of Anatomy in the Louisville Medical Institute.*

Solon Borland, M. D., *Memphis, Tennessee.*

A. H. Buchanan, M. D. *Columbia, Tennessee.*

Charles Caldwell, M. D. *Professor of the Institutes of Medicine and Medical Jurisprudence in the Louisville Medical Institute.*

Samuel A. Cartwright, M. D. *Mississippi.*

A. Clapp, M. D. *New Albany, Indiana.*

Jedediah Cobb, M. D. *Professor of Anatomy in the Louisville Medical Institute.*

Thomas W. Colescot, M. D. *Louisville, Ky.*

John Esten Cooke, M. D. *Professor of the Theory and Practice of Medicine in the Louisville Medical Institute.*

Samuel D. Gross, M. D. *Professor of Surgery in the Louisville Medical Institute.*

John Hardin, M. D. *Munfordsville, Ky.*

John P. Harrison, M. D. *late Professor of Materia Medica, in the Cincinnati College.*

John F. Henry, M. D. *Bloomington Illinois*

Samuel P. Hildreth, M. D. *Marietta, Ohio.*

Samuel Hogg, M. D. *Nashville, Tennessee.*

M. Z. Kreider, M. D., *Lancaster, Ohio.*

Moses L. Linton, M. D. *Springfield, Kentucky.*

Henry Miller, M. D. *Professor of Obstetrics and the Diseases of Women and Children in the Louisville Medical Institute.*

John W. Monette, M. D. *Washington, Mississsippi.*

Samuel B. Richardson, M. D., *Lecturer on Anatomy and Surgery, &c, Louisville, Ky.*

John L. Riddell, M. D. *Professor of Chemistry in the Medical College of Louisiana.*

Landon C. Rives, M. D. *late Professor of Obstetrics in the Medical Department of the Cincinnati College.*

Charles W. Short, M. D. *Professor of Materia Medica in the Louisville Medical Institute.*

G. Troost, M. D. *Professor of Chemistry and Mineralogy in the University of Nashville.*

Amasa L. Trowbridge, M. D. *Professor of Surgery, Willoughby University, Ohio.*

John A. Warder, M. D. *Cincinnati, Ohio.*

William Wood, M. D. *Cincinnati, Ohio*

### BIBLIOGRAPHICAL NOTICES.

I. *Transactions of the Medical Society of the State of New York.* Vol. v.

To express our opinion of the present number of these Transactions, it is perhaps compliment enough to say, that it is not less replete in valuable matter than any which have preceded it. The leading article is the "Annual Address," on Inflammatory Fever, by Sumner Ely, M. D., President of the Society. There is besides, a paper on the Nervous System, by Dr. Davis; one on Ergot, by Dr. J. B. Beck; one on Aneurism, by Professor Portal, of the Royal University of Palermo; and one on Hereditary Diseases, by Dr. Haynes. There is also an appendix, containing an abstract of the proceedings of the Society at its annual session, which was held in February, 1841; a list of officers of county societies; several legislative documents; and the regulation of Medical fees, by the Superintendents of the poor.

Our limits will not permit any thing like an opinion of each individual paper above enumerated; and of Dr. Beck's observations on the use and abuse of Ergot, which we have transferred to our columns, we need hardly add that we think we cannot better subserve the interests of the profession in the West than by laying it before them.

II. *Twenty-fourth Annual Report of the state of the Insane Asylum at Frankfort, near Philadelphia.*

We received sometime since this report, but, by an accident, it was lost before we had the pleasure of reading it. We will add, however, that we have seen very favorable notices of it in several of our exchange journals. Dr. Earle, the resident Physician, has rendered himself very favorably known to the profession, by his labors for relieving and mitigating the condition of the Insane.

### RECEIPTS FOR THE MEDICAL JOURNAL,

*For the month ending June 30th,* 1841.

| | |
|---|---|
| Dr. J. M. Townes, Oakichicama, Miss., | $10 00 |
| G. W. Holbrook, Waupankonetta, Ohio, | 5 00 |
| J. S. Mulloy, Mulloys, Tenn., | 2 00 |
| I. T. Underwood, New Castle, Ky., | 5 00 |
| J. McDonald, Athens, Ala., | 5 00 |
| D. Williams, Mt. Pleasant, Ohio, | 5 00 |
| J. H. Buell, Williamsport, Ia., | 5 00 |
| J. W. Foulke, Chillicothe, Ohio, | 5 00 |
| S. S. Cowan, Aberfoil, Ala., | 10 00 |
| R. L. Gilbert, Warrior Bridge, Ala., | 5 00 |
| J. M. Irwin, Holmesville, Miss., | 10 00 |
| C. H. Jordan, Roxboro, N. C. | 5 00 |
| Shanks, Memphis, Tenn., | 10 00 |
| W. W. Thompson, Big Spring, Tenn., | 10 00 |
| W. H. Lee, Flemingsburg, Ky., | 5 00 |
| J. H. Sowill, Athens, Ala., | 5 00 |
| J. P. Hill, Mt. Sterling, Ala., | 5 00 |
| S. C. Bridgewater, Clinton College, Tenn., | 5 00 |
| A. M. Debow, Hartsville, Tenn., | 5 00 |
| T. S. Head, Meadville, Miss., | 5 00 |
| J. A. Blakemore, Gallatin, Tenn., | 5 00 |
| J. M. Irwine, Mt. Sterling, Ill., | 5 00 |
| D. McFarland, Elizabeth, Ark., (discount $5,) | 10 00 |
| Wishard, Far West, Ia., | 5 00 |
| R. B. Harper, Portersville, Tenn., | 5 00 |

☞As the number of this Journal for March, 1841, is exhausted, we shall be much indebted to any one who may return us that number.

☞Postmasters and agents are requested to return us *all* the Nos. of this year, and the 1st volume of 1840, which have not been taken out by subscribers and are lying dead in their offices.

PRENTICE & WEISSINGER.

---

The Western Journal of Medicine and Surgery is published monthly by the undersigned, at the corner of Main and Fifth streets, Louisville, at $5 per annum, payable in advance. Each number contains from 80 to 84 pages making two volumes in the year of about 500 pages.

Contributions to its pages by the Physicians of the Valley of the Mississippi, addressed to the Editors, are respectfully solicited.

Letters on business, to be addressed, postage paid, to the publishers.

☞Postmasters, by the regulations of the Postoffice Department, will frank letters containing subscription money, and all remittances so franked are at the risk of the publishers.

*July* 25, 1840. PRENTICE & WEISSINGER.

